# Supplementary material for: Are carbon emissions trading and green financial instruments synergistic? -Comprehensive quantitative research based on content analysis
Source: PLoS One. 2024 Mar 7;19(3):e0298601. doi: 10.1371/journal.pone.0298601 (PMC10919601; doi:10.1371/journal.pone.0298601)
Supplement: S1 Data — (ZIP) [file pone.0298601.s001.zip › Data/地级市Z_F工作报告.zip!/╣π╬≈/╖└│╟╕█╩╨/└┤╫╘╣1⁄2╓┌║┼í╛┬φ┐╦╩2╛▌═°í┐_2006.docx]

各位代表：

现在，我代表市人民政府，向大会作关于防城港市国民经济和社会发展第十一个五年规划纲要的报告，请各位代表连同《纲要(草案)》一并审议，并请各位政协委员和其他列席人员提出意见。

“十五”时期工作回顾

“十五”时期，是我市建市以来发展最好的时期。全市经济发展、政治稳定、民族团结、边疆巩固、社会秩序良好、人民安居乐业、各项事业进步。五年来，我们在市委的正确领导下，在市人大及其常委会的监督和市政协的支持下，团结带领全市各族人民，围绕“兴港、强市、富民”和全面建设小康社会目标，按照“大港口、大通道、大工业、大开放、大发展”的思路，加快推进改革开放和现代化建设，圆满完成了国民经济和社会发展第十个五年计划。

综合经济实力增强，经济运行质量提高。初步统计，全市生产总值由2000年的57.51亿元增加到2005年的93.24亿元，年均增长11.5%（可比价，下同）；财政收入由2000年的4.13亿元增加到2005年的8.04亿元，年均增长14.2 %。“十五”期末，全市人均生产总值达到1460.00美元，人均财政收入达到1007.00元，“双过千”目标顺利实现。“十五”期间，全社会固定资产投资完成126.62亿元，年均增长23.0%；产业结构进一步优化，一、二、三次产业比重由“九五”期末的35:27:38调整为“十五”期末的24:35:41。旅游业稳步发展，房地产业明显升温。社会消费品零售总额由“九五”期末的14.58亿元增加到“十五”期末的22.75 亿元，年均增长9.3%。2005年全市固定电话用户26万户，互联网用户7.3万户，比“九五”期末分别增长2.8倍和7.5倍；金融机构存款余额80.62亿元，贷款余额43.34亿元，年均分别增长12.5％和8.3%。

港口发展跃上新台阶，核心竞争力增强。“十五”期间，累计投入15.85亿元用于加快港口建设。建成11、12号两个5万吨级泊位和5万吨级航道，新增吞吐能力460万吨；20万吨级泊位及配套航道投入试运营；13至17号5个万吨级以上泊位建设加快。“十五”期间，全市港口货物吞吐量8255万吨，年均增长16.6%。西南出海通道主门户地位得到巩固。

工业化进程加快，临港工业发展加速。“十五”期间，全市工业投资完成37.85亿元，其中：技改投资完成7.78亿元，分别是“九五”期间的6.0倍和1.9倍；工业总产值397.27亿元，年均增长24.4%；工业增加值100.31亿元，是“九五”期间的1.96倍。“十五”期间，全市规模以上工业由“九五”期间亏损2.15亿元转为盈利5.77亿元。“十五”期末工业对经济增长的贡献率比“九五”期末高9个百分点。

农业综合生产能力加强，县域经济稳步发展。“十五”期间，四个区、县（市）规模以上工业增加值19.30亿元，年均增长11.2%，初步形成制糖、制药、农特产品加工等支柱产业。农业综合生产能力得到加强。全市投入8.65亿元，对病险水库和海河堤进行除险加固；实施人畜饮水、灌区节水改造工程，解决了12.92万人的饮水难问题；农村道路、农网改造、沼气池、小水电站等建设给农民带来实惠。

重大项目相继落户，改革开放步伐加快。“十五”期间，沿海基础设施和重大项目建设取得新突破。防城港电厂开工建设，天宝（防城港）300万吨沥青项目启动；西湾跨海大桥、新兴220KV变电站扩建、茅岭110KV输变电、东兴国际展销中心等项目建成使用，江山至东兴一级公路、东兴至上思县际公路、防城港中油能源高纯复合LPG项目、万鑫钢铁、宏源浆纸等重大项目基本建成或投产；沙潭江至企沙一级公路、企沙工业区路网和供水管网、长歧干渠防渗加固扩能、白石牙水库、东湾110KV送变电等工程进展顺利；企沙千万吨级钢铁基地、广西白龙核电厂等项目前期工作取得重大进展。完成防城港务局政企分开和公司化改革，完成上思糖厂、防城糖厂、防城罐头厂等国有企业的改组改制，国有中小企业改制目标基本实现。农村税费改革试点工作取得实效；深化农村信用社改革试点工作顺利推进。“十五”期间，外贸进出口总额25.18亿美元，年均增长55.0%。边境贸易成交额完成132.06亿元，年均增长19.6%。

城乡基础设施改善，文明城市创建成效显著。“十五”期间，城市基础设施建设投入5.72亿元，比“九五”期间增加3.09亿元。建成西湾跨海大桥、西湾广场、十万吨水厂、垃圾无害化处理厂等市政设施。城镇化水平达到32.7%，比“九五”期间提高8.4个百分点。投入资金5749.85万元，新建和维修贫困村镇道路137条，饮水和水利工程352处；南屏瑶族乡基础设施建设大会战基本完成，港口区王府、上思县米安两个异地安置场建设通过自治区验收。防城、东兴、思阳、企沙、江平、那良、在妙等七个乡镇列为全区重点镇。

城乡居民生活水平明显提高，社会保障体系初步建立。“十五”期间，城乡居民收入年均分别增长8.3%和8.0%。大力推进“兴村富民”工程，“兴边富民”三年目标实现。深入开展减轻农民负担专项治理，健全城市居民最低生活保障制度和城乡社会救助体系。投入1000多万元建设五保村126个、敬老院11个，率先在全区实现乡乡（镇）有敬老院目标。落实了6600名五保人员的供养标准，建立了优抚对象定补自然增长机制。现役军人优待金纳入财政预算。建成市级及港口区、上思县社会福利院。农村医疗救助稳步推进。全市新增就业人数3.33万人，城镇登记失业率控制在3.5%以内。维护农民工合法权益，完成网上确认的农民工工资清欠工作。

各项社会事业取得新进步，精神文明和民主法制建设得到加强。“十五”期间，实施科技项目69项，开发工业新产品45项，推广农业先进技术66项。市高级中学、防城实验高中进入省级示范性高中行列；“两基”攻坚目标如期实现，农村办学条件明显改善；3.86万名贫困学生获得“两免一补”资助。建立了糖业、物流人才小高地和少数民族人才培训示范基地。精品文艺创作成效显著。完成50户以上自然村广播电视“村村通”工程，广播、电视人口覆盖率分别提高到90.8%和89.6%。公共卫生体系进一步健全，重大疾病预防控制工作取得新成效；新型农村合作医疗试点工作稳步推进。全民健身运动广泛开展，竞技体育获省级以上运动会奖牌150枚。人口自然增长率控制在8.9‰以下，出生人口性别比从123降至115。海洋管理和保护力度加大，完成东西湾海域综合整治；中国首个红树林国际示范区落户我市。在全区率先开展机关效能建设，推行“阳光政务”，取消行政审批事项960项，取消行政事业性收费项目129项，降低收费标准2405项。民族、统计、审计、气象、地震、档案、司法行政、人民防空、外事侨务、新闻出版、机关事务、地方志和未成年人保护、老年人、残疾人等工作都取得新成效。工会、共青团、妇联等群团组织在经济社会发展中发挥了积极作用。

精神文明和民主法制建设进一步加强。以创建“边海文明示范带”等活动为载体，切实加强思想道德和文化建设。获全国文明单位（村）6个，获自治区文明单位（村）35个，获自治区文明小区和文明庭院45个。“四五”普法全面完成。打击走私工作取得新成效，反走私综合治理工作经验被全国推广。安全生产工作得到加强，各项指标均控制在自治区下达的指标以内。“双拥”工作取得新成绩，荣获全国、全区双拥模范城称号。

自觉接受市人大和市政协监督，广泛听取社会各方面的意见和建议，人大代表建议、政协委员提案和社会各界来信来访的办理水平有所提高。行政许可法深入实施，依法行政逐步推进，行政效能进一步增强。保持共产党员先进性教育活动有效开展。廉政建设和反腐败斗争有序推进。

各位代表！刚刚过去的2005年，我市改革开放和现代化建设取得重大进展，经济社会发展加速，圆满完成了市三届人大六次会议审议通过的各项目标任务。初步统计，全年GDP完成93.24亿元，增长13.3%；全部工业总产值118.20亿元，增长25.8%；财政收入8.04亿元，增长18.6%；全社会固定资产投资42.96亿元，增长41.3%；全市港口货物吞吐量2377万吨（其中：防城港货物吞吐量2006万吨），增长23.2%；城镇居民可支配收入7248.00元，农民人均纯收入2704.00元，分别增长14.6%和7.4%；社会消费品零售总额22.75亿元，增长12.2%；外贸进出口总额8.50亿美元，增长14.9%；实际利用外资有较大幅度增长；人口自然增长率、城镇登记失业率以及居民消费价格指数均控制在计划以内。

各位代表！“十五”期间，在发生台风、洪涝、干旱等严重自然灾害以及受非典、禽流感疫情影响的情况下，我市能够取得这样的成绩是来之不易的。这是自治区党委、自治区人民政府和市委正确领导的结果；是市人大有效监督和市政协大力支持的结果；是全市各族人民共同努力的结果；是中直、区直单位和驻港部队支持帮助的结果。在此，我代表市人民政府，向全市广大干部群众，向驻我市解放军、武警官兵，向各民主党派、工商联、无党派人士、人民团体，向所有关心、支持我市改革开放和现代化建设的各界人士，表示衷心的感谢和崇高的敬意！

回顾“十五”时期不平凡的历程，我们深深地体会到：必须坚持以邓小平理论和“三个代表”重要思想为指导，紧紧围绕加快发展这个第一要务；必须贯彻和落实科学发展观，加快转变经济增长方式，不断提高经济增长质量和效益，促进经济社会全面、协调、可持续发展；必须深化改革，扩大开放，加强区域合作；必须紧紧抓住影响全市经济社会发展全局的重点、关键和薄弱环节进行合力攻坚，特别是要突出抓好重大项目建设，用发展的办法解决前进中的困难和问题；必须坚持以人为本，关注民生、体察民意，真心实意为群众办实事，千方百计为群众解难事，始终把实现好、维护好、发展好人民群众的根本利益作为工作的出发点和落脚点；必须正确处理好改革、发展、稳定的关系，维护社会稳定，促进社会和谐。

在肯定成绩、总结经验的同时，我们必须清醒地认识到，我市经济社会发展中仍然面临不少困难和问题。主要是：经济总量小，总体实力不强，工业化水平不高；农业基础设施建设滞后，抵御自然灾害能力比较脆弱；城市规模小，城镇化水平较低；财政比较困难，经费保障能力不强，社会事业发展滞后；一些事关人民群众切身利益的问题还没有得到很好的解决，人民生活总体上还不富裕；人才比较缺乏，成为制约经济社会发展的瓶颈；政府工作还有不少薄弱环节，行政效能有待提高。对于这些问题，我们将高度重视，采取切实有效措施，加快发展、深化改革、强化管理，努力加以解决。

“十一五”时期主要任务

“十一五”是我市“打牢基础、奋力爬坡、率先发展”的关键时期。我们一定要准确和科学判断形势，进一步增强加快发展的使命感、责任感和紧迫感，牢牢把握重大战略机遇期，积极应对各种挑战，努力开创我市改革开放和现代化建设的新局面。

根据市委三届五次全会通过的《建议》，我市国民经济和社会发展“十一五”规划总的指导思想是：坚持以邓小平理论和“三个代表”重要思想为指导，认真贯彻党的十六届五中全会、自治区党委八届六次全会和市委三届五次全会精神，紧密结合我市实际，全面落实科学发展观，抓住中国——东盟自由贸易区和“两廊一圈”建设步伐加快、“泛珠三角”区域合作加强以及自治区加快北部湾经济区发展等重大机遇，坚定不移地按照“大港口、大通道、大工业、大开放、大发展”的思路谋发展、抓发展、促发展；突出加强基础设施建设，统筹港口、工业和城市的发展，加快建设社会主义新农村，大力发展现代服务业，深化改革，扩大开放，不断提升经济综合实力和城市竞争力；加快构建和谐社会的步伐，重视社会事业发展，完善社会管理，维护边海防稳定，提高城乡居民生活水平；力争通过五年左右的努力，率先崛起北部湾经济区的港口龙头，率先建成广西新兴的工业强市，率先在全区实现小康社会的目标。

根据以上指导思想，“十一五”期间，我市国民经济和社会发展的总体目标是：继续保持经济较快发展的良好势头，在优化结构、提高效益和降低消耗的前提下，实现人均生产总值比2000年翻两番；经济增长的质量和效益进一步提高，财政实力明显增强，万元生产总值能耗比“十五”期末明显降低；港口大型化、专业化、现代化和国际化水平有较大提高，核心竞争能力增强，西南出海通道主门户的地位进一步巩固；工业化进程明显加快，“八大”工业体系基本形成，“四大”支柱产业初步确立，一批竞争能力强的优势企业逐步形成；城镇化水平明显提升，市政设施有较大改善，把我市初步建成环北部湾的区域性中心城市；以改革为动力，实施开放带动战略；增加就业岗位，健全社会保障体系，千方百计增加城乡居民收入；加快交通基础设施建设，努力改善投资环境；大力发展教育、文化、卫生、广播电视等事业。

“十一五”时期，我市国民经济和社会发展的主要目标是：生产总值年均增长20%左右（按可比价），人均生产总值年均增长17%左右（按可比价），“十一五”期末，人均生产总值超3000美元，率先在全区实现小康社会的目标；港口吞吐能力年均增长30%左右,2010年，港口吞吐能力突破6000万吨，率先崛起北部湾经济区的港口龙头；工业总产值年均增长32%左右；财政收入年均增长26%左右；外贸进出口总额年均增长20%左右；全社会固定资产投资年均增长36%左右；城镇化水平提高到45%以上；万元生产总值能耗比“十五”期末降低10%左右；城镇居民人均可支配收入年均增长9%，农民人均纯收入年均增长8%；城镇登记失业率控制在5%以内；城镇新增就业5万人左右，农村劳动力转移6万人左右；贫困地区人口基本脱贫；农村社会保障体系初步建立；城市基本养老、失业保险和基本医疗保险覆盖率分别提高到90%、85%和90%以上。

按照市委的总体要求，在编制《纲要（草案）》过程中，我们始终注意把握以下几点：一是根据党的十六届五中全会、自治区党委八届六次全会和市委三届五次全会精神，紧紧结合我市实际情况编制；二是既体现新形势的要求，又保持经济社会发展中长期规划的连续性；三是注意纲要与经济社会发展有关具体规划的相互衔接；四是既体现事关经济社会发展全局的总体部署，又突出重点、相互兼顾；五是努力使纲要具有可操作性。

《纲要（草案）》提出“十一五”期间的主要奋斗目标和总体要求是积极的，也是留有余地的，经过努力是完全可以实现的。在实际执行过程中，必须坚持以科学发展观统领经济社会发展全局；必须继续坚持“五大”发展思路和过去行之有效的好做法、好经验，促进港口、工业和城市的联动发展；必须坚持“产业强市、工业先行”的原则，在扩大经济总量、增强综合经济实力上狠下功夫；必须进一步扩大对外开放，大力发展开放型经济；必须坚持以人为本，切实提高人民群众生活水平；必须建设一支高素质的人才队伍，为加快经济社会发展提供坚实的人才保障；必须正确处理改革、发展、稳定的关系，促进社会和谐；必须牢固树立全市“一盘棋”思想，形成加快发展的强大合力。

下面，我着重报告《纲要（草案）》中的几个重大问题：

一、崛起北部湾经济区的港口龙头

港口是我市最大的资源优势，对大工业的布局起着决定性的作用。《纲要（草案）》提出，要加快港口大泊位、深航道及配套设施建设。“十一五”期末，全市港口货物吞吐能力突破6000万吨，率先崛起北部湾经济区的港口龙头，为实现亿吨大港目标打好基础，逐步把防城港建成环北部湾亿吨大港。在抓好在建泊位的同时，建设18至22号、501和502号泊位、防城港电厂码头，争取开工建设企沙钢铁基地配套泊位、20万吨级航道、东湾和西湾10万吨级航道，创造条件建设30万吨级铁矿石泊位和30万吨级油泊位。到2010年，防城港吞吐能力基本适应大西南和泛珠江区域货物集散疏运、货物中转贸易及我市发展工业的需要，把我市港口建成区域性的大型枢纽港、专业化工业港、现代化和国际化商贸港。

二、建设广西新兴的工业强市

加快推进工业化，事关我市发展大局。《纲要（草案）》强调，要坚定不移地把工业放在优先发展的位置，率先建成广西新兴的工业强市。“十一五”期间，要依托大港口优势，吸引一批关联度大、产业链长的大型临港工业落户，力争在引进投资超百亿元的特大型工业项目上实现新突破，打造广西钢铁、能源工业基地。依托大工业项目，大力发展配套产业，延伸大型工业产业链。重点抓好企沙·东湾工业区、江平工业区和东兴边境经济合作区、企沙千万吨级钢铁基地项目、广西白龙核电厂建设。加快发展面向中国——东盟市场的出口加工工业。不断发展壮大县域工业。大力发展特色海洋工业产业。加快“八大”工业体系和“四大”支柱产业建设，重点扶持一批核心企业，并将其培育成为年销售收入超30亿元、50亿元乃至100亿元的强优工业企业。

三、加快建设社会主义新农村

建设社会主义新农村，是统筹城乡发展的重大战略决策。《纲要（草案）》提出，要按照“生产发展、生活宽裕、乡风文明、村容整洁、管理民主”的要求，建设社会主义新农村。要强化“三农”工作，逐步实现农业产业化、农村城镇化、农民市民化目标。在保证粮食安全前提下，下大力气调整种植结构，大力发展特色农业；进一步调整畜牧业结构，加快发展草食动物；大力发展养捕结合以养为主的海洋渔业。积极培育“香、甜、苦、鲜”等特色产业，加快推进海产品、中草药等特色农产品生产加工基地建设，培育和扶持一批销售收入超亿元的龙头企业，推进农业产业化进程。加快社会化服务体系和农村公共设施建设。加大农村劳动力技能培训力度，扩大农村劳动力转移和输出规模。深化农村综合改革，巩固农村税费改革成果，切实减轻农民负担。

四、构建区域性商贸物流旅游中心

《纲要（草案）》强调，要大力发展以商贸业、物流业、旅游业、新型服务业为重点的现代服务业，逐步建成大西南、环北部湾和中国——东盟区域性的商贸中心、物流中心、旅游中心。大力发展以商贸业、中转服务业、出口加工贸易、边境贸易为重点的商贸服务业，基本形成布局合理、门类齐全、供应渠道畅通的商品市场网络。大力发展以大西南、中国——东盟市场和为我市工业服务为重点的物流业。着力打造上山下海出国特色旅游业。

五、建设环北部湾区域性中心城市

城镇化水平是衡量现代化的重要标志之一。《纲要（草案）》提出，要抓住自治区规划建设南北钦防城市群和北部湾经济区的重大战略机遇，按照“循序渐进、节约土地、集约发展、合理布局”的要求，统筹规划城镇布局与建设。突出发展城市中心区和边境城区，加快开发新城区，集约发展县城和小城镇，培育形成城镇群带，逐步把我市建设成为环北部湾集港口、工业、商贸、旅游为一体的区域性中心城市。坚持规划先行的原则，高起点地做好城市规划。突出以两区（城市中心区、东兴城区）、三岛（渔氵万岛、企沙半岛、江山半岛）为重点的城市化建设，逐步形成以城市中心区为核心，沟通防城、港口，连接东兴的城市格局。同时，统筹安排旧城改造和新城区开发，提高城市品位。坚持走以城带乡、以乡促城、城乡互动的城镇化发展之路，加快推进重点乡镇的规划与建设。

六、优化空间区域发展布局

促进区域协调发展是落实科学发展观、全面建设小康社会的重大战略任务。《纲要（草案）》把我市的功能经济区划分为“一带、三区”（沿海沿边经济带、中心经济区、边境经济区、十万大山经济区），提出各经济区（带）要结合产业的布局与发展，统筹规划布局城镇建设，逐步形成以沿海沿边城镇带、中心城镇带为核心的城镇体系。按照统筹区域和城镇协调发展的原则，加快崛起沿海沿边经济带，沿沙潭江至企沙一级公路和江山至东兴一级公路展开产业布局，重点发展临海临港临边工业，建成企沙至东兴沿海沿边经济带，使之成为中国连接东盟的重要经济走廊。加快中心经济区、边境经济区、十万大山经济区建设，促进区域和城乡协调发展。

七、加快推进基础设施建设

为适应加快发展的需要，《纲要（草案）》提出，要按照“统筹规划、加快建设、适度超前、完善网络、健全体系、提高能力、适应发展”的原则，加强交通、水利、能源、信息化等基础设施建设。交通方面，抓紧开工建设防城南站至企沙铁路，重点推进江山至白龙二级公路、企沙千万吨级钢铁基地项目配套路网、玉罗岭至李子潭一级公路、东湾跨海大桥及配套公路、中越北仑河二桥及配套公路、防城至上思二级公路的建设，实现区、县（市）通二级以上公路，全部乡镇通油路，大部分行政村通等级公路；创造条件开工建设防城至东兴连接越南铁路。口岸方面，重点抓好防城港、企沙、东兴、峒中和里火口岸基础设施建设。水利方面，要加快防汛抗旱减灾体系建设和水资源保护与开发，满足城市和工农业用水；加强水土保持及生态环境等工程建设。能源方面，重点推进防城港电厂建设和投产；力争早日开工建设广西白龙核电厂；建设防城港500KV变电站和防城、东兴、上思220KV输变电工程。信息化方面，重点建设电子政务工程，加快政府信息化，推进公共领域、社区和家庭信息化。

八、深化改革和扩大开放

深化改革、扩大开放是加快发展的强大动力。《纲要（草案）》强调，全面深化经济政治社会各领域的改革，加快形成落实科学发展观、促进经济增长方式转变、实现全面协调可持续发展的新体制。深化经济体制、财税体制和行政管理体制改革；改善非公有经济体制环境；健全要素市场体系。《纲要（草案）》强调，实施全方位、多层次、宽领域、互利共赢的开放战略，以开放促合作，以合作促发展。优化出口商品结构和市场结构，积极开拓市场特别是东盟市场。继续扩大一般贸易和加工贸易，大力发展服务贸易和技术贸易。优化外贸经营主体结构，扶持培育出口龙头企业，提高口岸通关效率。加大招商引资力度，不断扩大利用外资规模。积极参与区域合作，主动融入“泛珠三角”和东盟经济圈，推进东兴——芒街跨国经济合作区建设。

九、加强科技和人才队伍建设

科技和人才是经济社会发展的关键因素。《纲要（草案）》提出，要加快推进科技进步和人才队伍建设，为我市经济社会发展提供强有力的科技和人才支撑。适应我市发展大港口、大工业和城市建设需要，按照“自主创新、重点跨越、支撑发展、引领未来”的要求，加快推进科技进步与创新。积极推广农业现代配套技术，运用高新技术和先进适用技术改造传统企业。重点实施科技兴企、星火富民、农产品深加工、中药现代化和科技自主创新能力提升工程，力争在海洋生物、品种改良、海水养殖、制药制糖、食品加工和节能降耗等领域取得新突破。牢固树立人才资源是第一资源的观念，着力抓好培养、吸引、用好人才三个环节，加强人才资源整合开发，全面提升人才队伍素质，为加快发展提供人才智力保障。重点加强农村科技、教育、卫生、生产、经营等实用人才队伍建设，着力培养技术技能型、知识技能型和复合技能型人才。依托物流、粮油、制糖、能源、冶金等重点产业和重点项目，加大人才小高地建设力度，吸引和集聚一批高层次专业技术和企业经营管理人才，适应经济社会发展需要。

十 、加快构建和谐社会

《纲要（草案）》强调，要按照“以人为本、关注民生、优化环境、保持稳定、安居乐业、和谐进步”的要求，加快推进“富裕文明、碧海蓝天、安定有序、人民康乐”的和谐社会建设。坚持节约资源和环境保护的基本国策，加强资源综合利用，大力发展循环经济；加强环境保护和生态建设，重点加强对十万大山水源林生态区的保护和十万大山国家森林公园、北仑河红树林、防城金花茶等国家级自然保护区的保护利用；全面推进国家生态示范区建设，争取列入国家生态城市建设试点，努力把我市建成国家级生态城市。

加大教育投入，不断改善农村办学条件，巩固发展“两基”成果；积极推进素质教育，提高教育质量；大力发展职业教育，整合职业教育资源。加强文化阵地建设，大力发展文化产业。深入开展全民健身运动，不断增强人民体质。加大文化、体育等公益基础设施投入，满足人民群众文体生活需要。加快发展卫生事业，重点创建一至两所“三甲”医院，加强县级医院和乡级卫生院的建设，全面推行新型农村合作医疗；加强疾病预防控制体系和基层预防保健机构建设，抓好重大传染病防治。认真落实人口与计划生育目标管理责任制，稳定低生育水平；完善城乡人口和计划生育管理网络，加强流动人口计划生育管理，提高人口素质。建立健全应对突发公共事件的应急机制和防控体系，提高处置突发公共事件能力。

加大政府对民生事业的投入，促进和谐社会的构建。加快推进以贫困地区为重点的“兴村富民”工程，大力推进以水、电、路为重点的农村基础设施建设，全面完成90个贫困村的整村推进任务。千方百计扩大就业，完善对困难群众的就业援助制度；建立城乡统一劳动市场，促进劳动力的有序流动。巩固“两个确保”工作成果，完善职工基本养老、基本医疗和失业、工伤、生育等社会保障制度；增加财政对社会保障投入，多渠道筹措社会保障基金；完善城市居民最低生活保障制度，认真解决进城务工人员社会保障问题；抓好失地农民的培训和就业安排。完善社会救助体系和优抚保障机制，建立健全城乡医疗救助、住房救助制度。加强社会福利服务网络建设。认真落实双拥优抚安置政策。建立健全与经济发展相适应的收入增长机制，增加城乡居民收入。加强社会稳定工作，努力构建稳定有序的社会安全环境。加强安全生产监督管理，注重安全生产设施建设，确保人民群众生命财产安全。

二○○六年工作安排

2006年是“十一五”规划的开局之年，也是实现“三个率先”的起步之年。做好今年的工作，对于巩固和发展“十五”时期取得的成就，保持经济社会发展的良好势头，顺利完成“十一五”规划的各项任务，具有重大意义。

根据市委三届五次全会提出的“十一五”规划指导思想，今年我市经济社会发展的主要目标是：GDP增长14%左右，工业总产值增长25%，全社会固定资产投资增长45%，财政收入增长16%，港口货物吞吐量增长12%，社会消费品零售总额增长12%，外贸进出口总额增长16%，实际利用外资增长30%，城镇居民人均可支配收入增长8%，农民人均纯收入增长7%，城镇登记失业率控制在4%以内，人口自然增长率控制在10‰以内。

为实现上述经济社会发展目标，必须重点抓好以下几个方面的工作：

一、大力推进重大项目建设，增强经济发展后劲

项目建设是发展的物质载体，是改善发展环境、夯实发展基础、增强发展后劲的关键。要紧紧抓住自治区推进北部湾经济区规划建设的大好机遇，按照“完善设施、构建网络、健全体系、发挥能力”的要求，进一步加大项目建设力度。

加快重大项目建设。今年计划安排全社会固定资产投资62.3亿元，拟安排重大项目45项，投资47亿元左右。要想方设法确保任务的完成。加快建设沙潭江至企沙一级公路、白石牙水库、企沙工业区供水管网、长歧干渠防渗加固扩能等沿海基础设施建设大会战项目，确保今年8月全面建成。上思至崇左二级公路年内全线贯通，建成防城城区防洪排涝一期工程和上思县那板灌区那豆渡槽工程。力争开工建设西南明珠国际城、广西巨星影视娱乐城、市公务员小区、市道路运输信息管理指挥中心、市第一汽车客运中心、港口区黄泥潭海堤工程、东兴市界河水毁修复工程、上思县小水电开发、防城港500KV变电站、企沙渔港改造等项目。

加强项目前期工作。重点推进企沙千万吨级钢铁基地项目、广西白龙核电厂、防城南站至企沙铁路、钢铁基地配套交通路网、江山至白龙二级公路、东湾西湾航道工程、中越北仑河二桥及配套公路、东湾工业区配套道路、皇帝岭至针鱼岭滨海二级公路、防城至茅岭二级公路、东湾跨海大桥、大垌水库等项目的前期工作。

健全项目建设机制。强化项目建设目标责任制，完善市领导挂钩联系重大项目建设制度，形成主要领导负总责，有关部门具体负责、相关部门积极配合的项目建设机制。加大检查监督力度，及时解决项目建设中的问题。抓住投融资这个重点，创新投融资机制，拓宽投融资渠道。加强项目资金管理，严格工程监理，力创优良工程。

二、大力推进港工互动，突出发展港口和临港工业

按照“以港引工、以工促港、港工互动”的思路，以大港口为依托，以招商引资为突破口，以优化投资环境为保障，加快构建“八大”工业体系和“四大”支柱产业。

加大港口建设力度。完成20万吨级泊位及配套航道建设。抓紧13至17号泊位建设，完成码头水工、后方堆场及设备安装；加快吹填11、12号泊位后场和矿石专用堆场；加快5万吨级液体化工码头建设；做好18至22号泊位、30万吨级油码头、煤炭专用码头等项目前期工作。重点抓好防城港粮糖物流中心、万鑫物流等项目建设，做好企沙·东湾工业区物流中心、里火口岸国际物流中心的前期工作，培育一批主营业务突出、竞争力强的物流龙头企业。

突出发展临港工业。遵循“产业强市、工业先行”的原则，重点推进一批临港大工业项目建设。宏源浆纸、防城港中油能源高纯复合LPG等项目要建成投产；防城港电厂要加快建设，天宝（防城港）300万吨沥青项目要正式开工。加快广丰硫酸亚锡厂等项目建设。按照“大项目——产业链——产业集群——制造业基地”的思路，大力扶持和推动相关配套产业和企业集群发展，拉长产业链，做大配套工业。在加快企沙·东湾工业区建设的同时，推进东兴江平，港口渔洲城、公车，防城河西、茅岭和上思城西等县域工业区的规划建设。大力扶持强优企业。加大对大海粮油、新海油脂、万鑫钢铁等重点骨干企业的扶持培育，在煤电油运供给、项目申报、技改贴息、用地用海等方面实行倾斜，激励企业向大公司、大集团发展。

大力发展海洋经济。科学制定海洋经济发展规划，综合开发和合理利用海洋资源。在发展海水养殖和捕捞的同时，培育海产品加工龙头企业，促进海产品加工工业的发展；积极发展海洋生物和海洋化工产业；依托港口和滨海旅游资源，大力发展港口运输和滨海旅游业。

三、大力推进社会主义新农村建设，保持农业和农村经济稳步发展

大力发展特色农业。稳定粮食播种面积，提高高产优质品种比例和粮食生产科技含量。加快糖料蔗生产良种化，提高甘蔗单产和含糖量。全面实施“优果工程”，重点发展名特优水果。大力建设速丰林和名特优经济林基地，新增造林9万亩，完成肉桂、八角经济林低改10万亩。着力打造林浆纸和木材综合利用、林产化工、生态旅游等支柱产业。大力扶持良种畜禽生产，完成9个养殖小区示范点建设。

加快农业产业化进程。围绕优势产品和产业，做强做大岳泰饲料、海世通、富新科技等农业产业化龙头企业。以蔗糖、香料、浆纸、水产等龙头企业为依托，建立一批种养致富示范基地，带动农民增收。建立健全农业社会化服务体系，争取每个乡镇都培育发展2—3个行业协会和农村经济协会，提高农民进入市场的组织化程度。

加强农村基础设施建设。完成除险加固病险水库19座，建成海河堤防、渠道硬化16公里，修复农田水利水毁工程146处，完成农村人饮国债项目32个；扩建和续建农村公路10条，建成乡镇客运站4个，便民码头6个。加快农村电力、通信、教育、文化、卫生等基础设施建设。

推进“兴村富民”工程。围绕整村推进五大类28项量化指标，扎实开展“兴村富民”工作，实现6500人脱贫。首批38个贫困村要通过自治区检查验收，同时做好第二批贫困村整村推进前期准备工作。加强科技培训，使大多数农民掌握一至两门致富实用技术。建立健全农村劳动力转移培训体系，提高农民综合素质，确保完成2000人培训任务，新增农村劳动力转移就业1万人。

四、大力推进城市化进程，培育区域经济增长极

加快城市基础设施建设。采取多元化、市场化办法筹措城市建设资金，上半年完成城市中心区主体建筑群和东西大道、南北大道、迎宾路、中心广场建设。中心区环路工程要全线开工建设；群星大道北延段、双拥路东段、凯乐园路西段道路及配套工程要动工兴建；力争开工建设市污水处理厂、市管道燃气项目和东兴市污水处理厂。加快城市道路、排水（排污）管道、街道路灯照明、城市园林绿化等配套设施建设。

做好城市规划和管理。统筹做好区域规划、城市规划和土地利用规划。重点做好城市和土地利用规划的修编。强化城市管理，采取政府特许经营权方式，逐步把污水处理、公共交通、环境卫生等市政公用事业推向市场。大力开展市容市貌及环境卫生综合整治工作，搞好城市绿化、亮化、净化和美化。抓好烂尾楼工程处置工作，确保年内全部完成。

抓好小城镇建设。推进企沙、江平、江山、峒中、在妙等重点乡镇建设，根据各镇建设规划做好基础设施配套，积极引导社会资金参与重点乡镇开发。加快实施“123”村镇规划工程，用一年左右时间完成建制镇总体规划编制任务，用两年左右时间完成未建制集镇总体规划编制任务，用三年左右时间完成主要村庄总体规划编制任务，企沙半岛和江山半岛所在村镇的规划年内要完成。

加快沿海沿边经济带开发建设。整合企沙至东兴沿海沿边经济带资源，大力发展优势特色产业，逐步建立起连片开发、联动推进、优势互补的经济带，促进沿线城区和村镇互动发展，将企沙至东兴沿海沿边经济带建设成为集交通运输、观光旅游、新型工业、现代农业、贸易加工等为一体，配套设施齐全、环境生态一流的国门边海通道经济带。

五、大力推进现代服务业，增强消费对经济增长的拉动力

做大做强旅游产业。坚持抓好市内游、边境游、入境游等工作，重点加快江山半岛旅游度假区、十万大山国家森林公园、京岛风景名胜区创4A建设步伐，继续办好十万大山原始森林旅游节。结合建设社会主义新农村，大力发展农业观光游。尽快恢复防城港至越南下龙湾海上旅游航线。大力推行所有权、经营权、管理权“三权”分离的景区经营管理模式，鼓励和吸纳社会资本投资兴办旅游业。

大力发展商贸业。完善商业网点规划布局，加大流通基础设施建设力度。推广连锁经营、特许经营、物流配送、电子商务等新型流通方式，提高流通业现代化、规范化和组织化水平。加快农村商业网点改造，努力构建城乡双向流通网络。拓展农村各类生活服务消费，建设以县城为中心、乡镇为重点、村社为基础的新型农村流通网络和服务网络。

积极发展服务业。大力支持银行、保险等金融服务业改革与发展，加强银政、银企合作。加快经济适用住房建设，促进房地产业健康发展。积极发展法律、会计、评估、社区服务等新兴服务业。

创造良好的消费环境。培育住房、汽车等消费热点，促进消费结构升级。积极发展消费信贷，促进个人信用消费。

六、大力推进对外开放，提高利用外资的质量和水平

突出抓好招商引资。抓住国际制造业转移的机遇，大力实施以制造业为主的产业链招商。充分利用中国——东盟博览会平台，认真组织参加自治区的重点招商活动，有计划、有目的、有针对性地组织小分队招商，突出招商重点。建立和完善招商引资项目库，加强与自治区招商项目对接。继续办好中越边境（东兴）商品交易会。认真做好项目跟踪落实和服务工作，及时协调解决项目进展过程中的困难和问题。落实有关鼓励政策，为企业提供法律、法规、政策、市场、资源及涉外信息等服务。

扩大对外贸易。优化出口商品结构，主动对接东盟，强化重点出口商品跟踪服务。鼓励加工贸易企业进行技术改造，开发技术含量高、附加值高的产品，打造我市出口产品品牌。鼓励外经贸企业积极利用外资，参与国际竞争，开拓出口创汇新渠道。

加强口岸建设。加快口岸基础设施建设，力争开工建设防城港、东兴联检大楼和国际客运站改造项目，建立口岸信息平台和视频监控，加强口岸之间合作，推动口岸向集约化、规模化和效益化方向发展。加快口岸综合管理现代化进程，提高口岸整体运作效率。

七、大力推进各项改革，为经济快速发展奠定体制保障

积极稳妥推进国企改革。推进企业国有产权改革，争取全面完成国有企业改革任务。特别是加快推进防城港务集团与香港远航集团、西班牙德佳德斯公司合作，努力开创引进战略投资者的新局面。

大力发展非公有制经济。坚持在政治上鼓励、政策上支持、方向上引导、法律上保护，加强与改进政府对非公有制企业的服务和监管，放宽市场准入，放手发展个体、私营等非公有制经济，提高非公有制经济占国民经济的比重。

深化投资体制改革。创新投融资机制，加强与金融机构合作，引进培育壮大承贷主体、开发主体和合作主体，盘活政府有形资产和无形资产，回收资金滚动发展基础设施和公益事业，逐步实现投融资主体多元化。对符合国家产业政策的重大项目，采取直接融资、收费质押、项目资产作价抵押、BOT等方式融资，多渠道解决建设资金不足问题。

加快财税体制改革。继续深化部门预算改革，在市本级全面实施综合部门预算。全面铺开市本级国库集中收付和政府非税收入收缴管理制度改革。完善政府采购制度，逐步将公共工程项目纳入政府采购管理。深化税收征管体制改革，加强税收征管和税源监控管理。

抓好农村综合改革。稳步推进乡镇机构、农村义务教育和县乡财政管理体制改革，巩固农村税费改革成果。进一步深化粮食流通体制改革，确保粮食安全。深化农村信用社改革试点工作，增强农村金融服务县域经济能力。创新水利经营管理机制，推进水利体制改革。

八、大力推进各项社会事业，促进社会和谐发展

加强就业和社会保障。培育和增加就业岗位，新增城镇就业8500人，城镇下岗失业人员实现再就业1350人，城镇登记失业率控制在4%以内。巩固“两个确保”，抓好社会保险扩面征缴。加大劳动监察力度，维护进城务工人员合法权益。继续抓好城市低保工作，探索农村低保路子，确保农村五保户和特困家庭基本生活保障。争取建设市备灾救灾指挥中心和市老年服务中心，进一步做好救灾救济工作。

切实抓好社会稳定和安全生产。大力推进“平安防城港”建设。加强防范和依法打击敌对势力，严厉打击贩毒、暴力、经济和侵财犯罪。继续实施反走私综合治理工程。进一步改进信访工作，积极预防、妥善处置群体性事件。强化安全生产责任制，完善安全监管网络体系，规范安全生产秩序，提高安全生产能力。

强化应急管理工作。把应急管理工作摆上更加重要位置，明确责任，整合资源，建立市突发事件应急指挥中心。尽快建立健全“统一指挥、反应灵敏、功能齐全、运转高效”的应急管理机制、预案体系，提高政府保障公共安全和处置突发事件的能力。

大力推进各项社会事业。巩固“两基”成果，落实“两免一补”等政策，加大对山区、贫困地区和少数民族聚居区的基础教育投入；大力发展职业教育，创办市职业教育中心。推进事业单位人事制度改革，加快实施人才小高地建设规划，有针对性地培训、引进各类人才。以提高自主创新能力为重点，启动中小企业创新科技服务网络；全面实施农村科技信息网络“乡乡通”项目，抓好“村村通”示范点建设。加快市文化艺术中心和市公共图书馆项目前期工作，挖掘京族等特色民族文化，积极申报国家级非物质文化遗产项目。继续实施20户以上通电自然村“村村通”广播电视工程，巩固、扩大“西新工程”建设成果，推进城市有线电视数字化整体转换。认真落实人口和计划生育基本国策，加快建立人口和计划生育工作新机制，落实人口与计划生育目标管理责任制。加快公共卫生体系建设，完成市传染病区、紧急医疗救援中心项目建设；加强卫生行业监管，提高公共卫生服务能力和质量；加强高致病性禽流感、艾滋病、结核病等重大传染病防治（控）工作；扩大新型农村合作医疗试点。推动全民健身，提高体育运动竞技水平。关心和支持老龄、妇女、儿童、残疾人工作。民族、侨务、统计、审计、外事、气象、档案、修志、新闻出版、机关事务、防震减灾等工作要取得新的成绩。

加强人居环境建设。抓紧制定建设节约型社会重点工作规划和实施方案，积极创建节约型城市、节约型政府、节约型企业、节约型社区。大力推进循环经济、清洁生产和节能降耗工作。在重点行业开展清洁生产示范，重点抓好一至两个清洁生产示范企业；认真开展ISO14000环境管理系列标准和环境标志认证工作。加强国土资源管理，统筹安排各类建设用地，盘活存量土地；加强海域使用管理与海洋环境保护，规范海域使用行为；坚决打击非法采矿活动，合理开发利用资源；抓好土地开发整理复垦工作，争取新增耕地突破5000亩。

九、大力推进精神文明和民主法制建设，为经济社会发展创造良好环境

加强精神文明建设。继续实施“边海文明示范带”创建工程，深入开展文明单位、文明村镇和“诚信防城港”等创建活动，树立良好国门形象。认真落实《公民道德建设实施纲要》，加强职业道德、社会公德、家庭美德教育和未成年人思想道德建设。以社区服务为龙头，推进文明社区建设。移风易俗，推进殡葬改革。

加强民主法制建设。自觉接受市人大及其常委会监督，坚持向市政协通报制度，充分发挥工会、共青团、妇联等群团组织的作用。巩固和扩大基层民主，完善村民自治、社区民主管理和企业管理制度，推进村务公开和厂务公开。加大依法治市力度，做好“五五”普法启动工作，加强法律援助，深入开展基层人民调解组织规范化建设。加大纠风治乱工作力度，切实维护群众利益。

再创双拥模范城。加强国防动员、人民防空、交通战备、边海防设施和民兵预备役建设。落实优抚政策，做好随军家属安置、部队子女入学入托等工作，推进双拥工作进工厂、进企业、进社区，促进双拥工作社会化，再创全国双拥模范城。

十、努力建设廉洁高效诚信政府

围绕“规范、高效、廉政、务实、民主、团结”的目标，加快政府职能转变，强化政府经济调节、市场监管、社会管理和公共服务职能，努力建设廉洁高效诚信政府，为实现“三个率先”提供有力保障。

加快政府职能转变，努力提高工作水平。认真学习新知识、研究新情况、发现新问题、把握新规律，不断提高落实科学发展观、驾驭市场经济和管理社会的能力。大力推进政府管理创新，由直接管理向间接调控转变，努力实现政府调控机制同社会协调机制互联、政府行政功能同社会自治功能互补、政府管理力量同社会调节力量互动。全面实施政务公开，加强电子政务建设，增强政府工作透明度，提高政府公信力，树立政府权威。规范政府行为，简化办事程序，强化执行能力，提高行政效率。

坚持求真务实，切实转变机关作风。各级领导干部要在思维方式、工作方法、工作作风和精神状态等方面，努力适应“三个率先”的需要。实干就是能力，落实就是水平。坚决反对形式主义，力戒浮躁之气，切实从繁文缛节中解脱出来，用更多的时间和精力谋发展，深入基层、深入群众、深入调查研究，努力办实事、解难题、抓落实、促发展。树立事业心、增强责任心、激发进取心，想大计、干大事、创大业，以业绩论英雄，体现出一种敢闯敢试、开拓创新的锐气；一种迎难而上、坚韧不拔的勇气；一种自我加压、争创一流的志气；一种全力以赴、奋发进取的士气!

全面推进依法行政，坚持科学民主决策。认真落实依法行政实施纲要和依法治市各项措施，确保令行禁止。完善行政责任追究制，健全行政权力运行监督制约机制。充分发扬民主，重大决策必须深入调查研究，广泛听取各方面的意见和建议，努力提高决策科学化、民主化水平。认真贯彻执行公务员法，不断增强公务员依法行政观念，提高依法行政水平，做到职权法定、依法行政、有效监督、高效便民。

加强政风建设，增强拒腐防变能力。坚持群众观念，以克服形式主义、官僚主义为着力点，以思想教育和制度建设为根本点，全面落实党风廉政建设责任制，进一步加强政风建设。建立健全教育、制度、监督并重的惩治和预防腐败体系，努力从源头上预防和遏制腐败；权为民所用，情为民所系，利为民所谋，一心一意谋发展，聚精会神搞建设，以良好的政府形象取信于民!

各位代表！回首“十五”，令人鼓舞；展望未来，催人奋进。让我们更加紧密地团结在以胡锦涛同志为总书记的党中央周围，高举邓小平理论和“三个代表”重要思想伟大旗帜，全面贯彻落实科学发展观，在市委的坚强领导下，振奋精神、坚定信心，团结一致、齐心协力，锐意进取、艰苦创业，为实现“十一五”规划的宏伟目标而努力奋斗！
